# Primary cutaneous epithelioid hemangioendothelioma of the temporal region: a case report

**DOI:** 10.11604/pamj.2025.52.165.50455

**Published:** 2025-12-17

**Authors:** Xinrong Chen, Yusa Chen, Yuehuan Bian

**Affiliations:** 1 Department of Dermatology, Zhuji People's Hospital of Zhejiang Province, Zhuji, China,; 2 Department of Pathology, Zhuji People's Hospital of Zhejiang Province, Zhuji, China

**Keywords:** Epithelioid hemangioendothelioma, primary cutaneous, temporal region, case report

## Abstract

Primary cutaneous epithelioid hemangioendothelioma (EHE) is an uncommon low-grade malignant vascular neoplasm that predominantly involves visceral organs, with primary manifestation in the skin, particularly in the head and neck region, being exceptionally rare. This report describes the case of a 70-year-old female presenting with a gradually progressive, intermittently ulcerated plaque on the left temple. Histopathological evaluation revealed characteristic features of EHE, which were further confirmed by strong immunohistochemical expression of endothelial markers CD31 and ERG. Comprehensive systemic assessment demonstrated no evidence of extracutaneous disease. The patient declined surgical intervention due to aesthetic concerns and has been maintained on follow-up every three months. No disease progression has been observed during the six-month follow-up period. This case highlights the importance of including cutaneous epithelioid hemangioendothelioma (EHE) in the differential diagnosis of persistent ulcerative facial lesions and underscores the value of early histopathological biopsy. Early, localized lesions can be completely excised surgically, achieving cure while optimally preserving facial aesthetics.

## Introduction

Epithelioid hemangioendothelioma (EHE) is an uncommon vascular neoplasm of borderline malignancy, with an estimated incidence of less than one per million [1]. Although it typically involves the liver, lungs, or bones, primary cutaneous EHE (cEHE) is exceedingly rare [2]. The limbs are the most frequently affected sites, with involvement of the temporal region being highly unusual [3]. Clinically, cEHE presents as solitary or multiple red-to-violaceous papules, nodules, or plaques that may ulcerate. Its nonspecific clinical and histological features often lead to diagnostic delay or misdiagnosis. We present a case of primary cEHE located in the temporal region to enhance clinical recognition, discuss the diagnostic approach, and review the therapeutic challenges associated with lesions in cosmetically sensitive areas.

## Patient and observation

**Patient information:** a 70-year-old Han Chinese woman presented to the dermatology department with a two-year history of a persistent, slowly enlarging skin lesion on her left temple. The lesion began as an asymptomatic dark red papule. Over the following months, it gradually increased in size, developed mild pruritus, and underwent cycles of ulceration and crusting. During this period, the patient presented on multiple occasions and was managed solely with local anti-inflammatory treatment. The cutaneous lesion showed no improvement and progressively enlarged. Her past medical history and family history were non-contributory.

**Figure 1:**
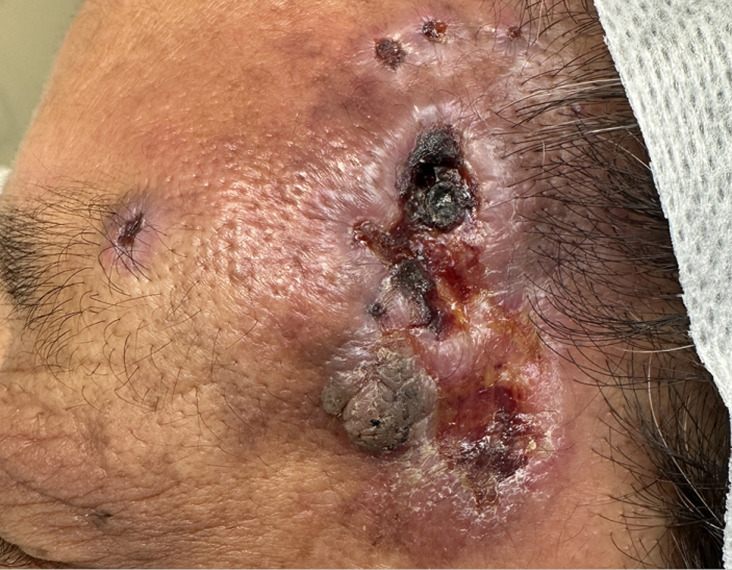
clinical presentation of primary cutaneous epithelioid hemangioendothelioma on the left temporal region, a dark red, irregularly shaped plaque with surface erosion and crusting is observed, a firm, brownish nodule is visible within the plaque

**Clinical findings:** physical examination revealed no systemic abnormalities. Dermatological inspection showed a dark red, irregularly shaped plaque measuring approximately 4 cm in diameter on the left temporal area. The plaque surface was partially eroded and covered with crust, and a firm, brownish nodule was observed within it (Figure 1). No regional lymphadenopathy was detected. Routine laboratory investigations, including complete blood count, liver and renal function tests, coagulation profile, and standard tumor markers (CEA, CA19-9), were within normal limits. Contrast-enhanced computed tomography (CT) of the chest and abdomen and magnetic resonance imaging (MRI) of the head were performed to exclude metastatic disease; both showed no evidence of internal organ involvement or underlying bone erosion.

**Timeline of current episode:** two years before, the patient first developed a rash in the temporal region; over this period, the patient was evaluated in the outpatient clinic on multiple occasions and was treated solely with topical anti-inflammatory agents; day 0-a local biopsy of the cutaneous lesion was performed; day 1-preliminary pathological examination raised suspicion for a vascular tumor and recommended immunohistochemical studies; day 5-immunohistochemistry results confirming the diagnosis of cEHE; day 7-declined the procedure due to aesthetic concerns and opted instead for ongoing surveillance with follow-up examinations scheduled every three months; 3 months later-follow-up, no change in the rash was observed; 6 months later-follow-up, no change in the rash was observed.

**Figure 2:**
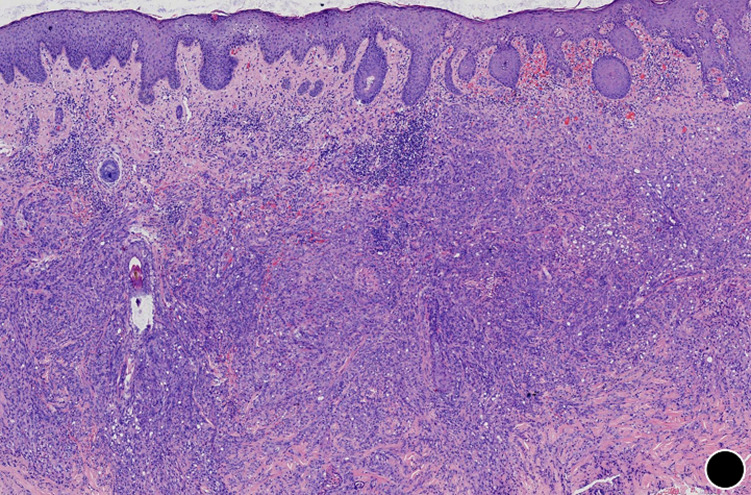
the tumor demonstrates an infiltrative growth pattern within the dermis (x50)

**Figure 3:**
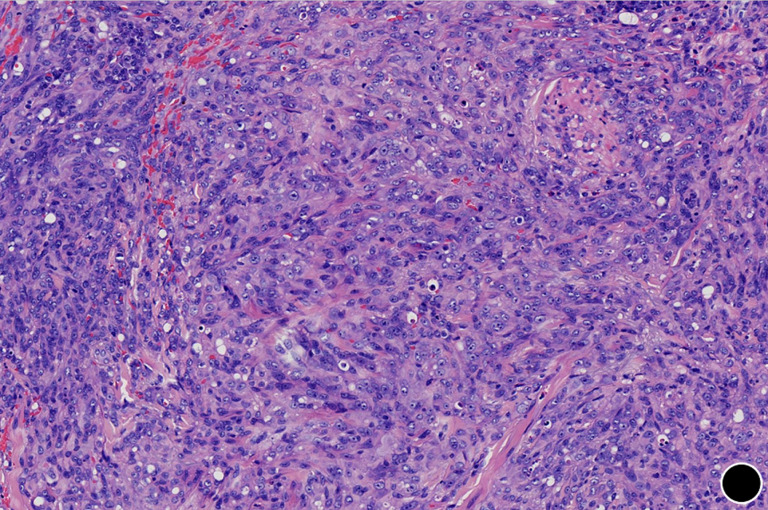
tumor cells are epithelioid and plump spindle-shaped, exhibiting mild nuclear atypia and eosinophilic cytoplasm (x100)

**Figure 4:**
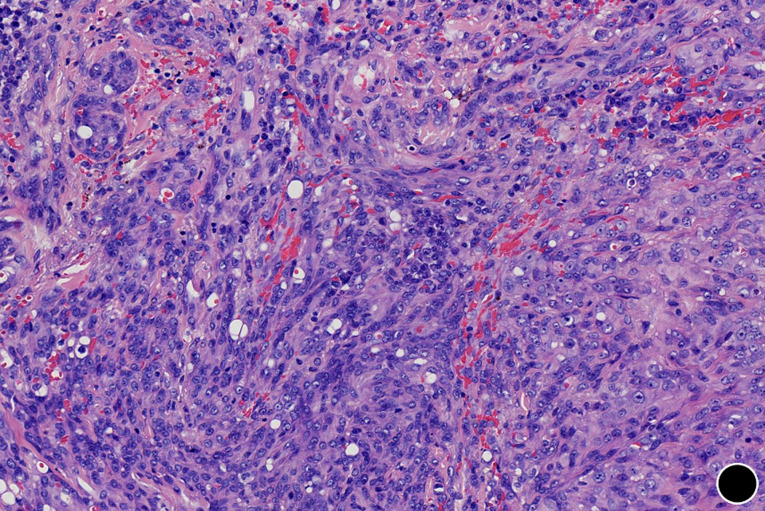
intracytoplasmic vacuoles containing red blood cells, representing primitive vascular lumina (x200)

**Figure 5:**
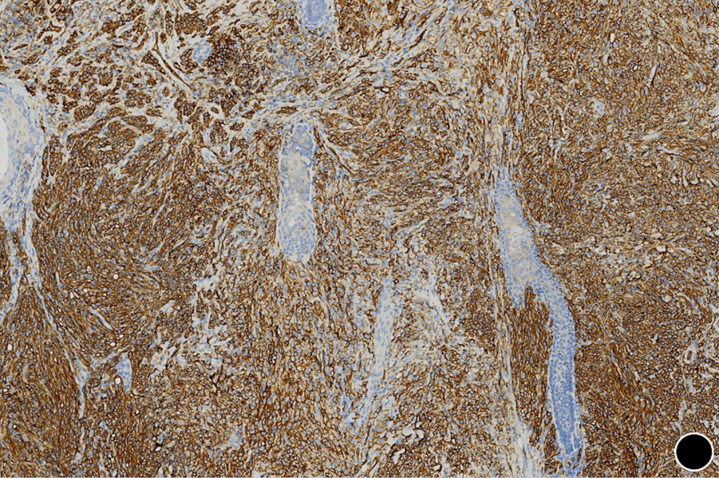
strong and diffuse membranous positivity for CD31 (x50)

**Figure 6:**
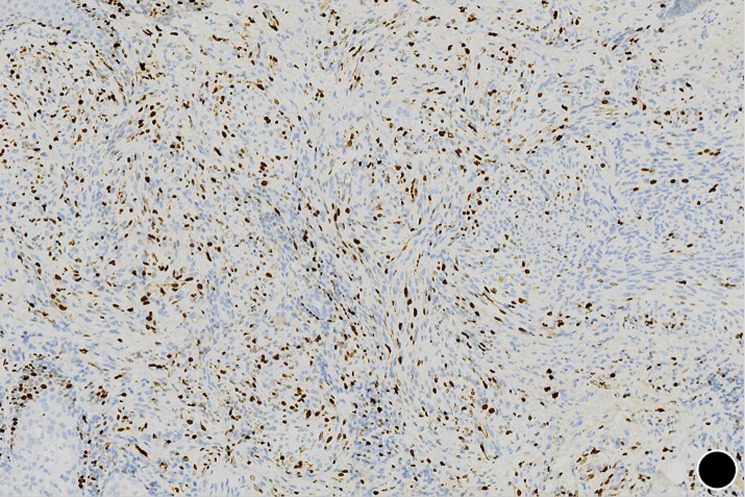
the case demonstrated a Ki-67 proliferation index of approximately 30% (x50)

**Diagnostic assessment:** routine laboratory studies, including complete blood count, liver and renal function panels, coagulation profile, and standard tumor markers, showed no significant abnormalities. An incisional biopsy was performed under local anaesthesia. Histopathological examination revealed an ill-defined, infiltrative dermal tumor arranged in cords and solid nests (Figure 2). The tumor was composed of epithelioid and plump spindle-shaped cells with mild nuclear atypia and eosinophilic cytoplasm (Figure 3). Nuclei were round to oval, with small, conspicuous nucleoli; mitotic figures were rare. A diagnostically critical finding was the presence of intracytoplasmic vacuoles, some of which contained intact red blood cells, representing primitive vascular lumina (Figure 4). The tumor stroma was focally myxoid. Immunohistochemical staining was decisive. The tumor cells exhibited strong and diffuse positivity for CD31 (Figure 5) and ERG, with focal positivity for CD34. The Ki-67 proliferation index was approximately 30% (Figure 6). The tumor cells were negative for cytokeratin (CK), CD68, TFE3, HMB45, BCL-2, S-100, smooth muscle actin (SMA), and desmin. This immunoprofile confirmed vascular endothelial differentiation and supported the diagnosis of primary cutaneous epithelioid hemangioendothelioma. Metastatic disease was ruled out by chest and abdominal CT scans as well as head MRI. Genetic testing was not performed due to platform limitations.

**Diagnosis:** primary cutaneous epithelioid hemangioendothelioma of the temporal region.

**Therapeutic interventions:** the patient presented on multiple occasions for local anti-inflammatory treatment, which proved ineffective, and the cutaneous lesions gradually enlarged. A histopathological biopsy was performed during the current evaluation, confirming the diagnosis. Given the extensive nature of the lesion, surgical intervention was declined by the patient due to aesthetic concerns. Follow-up observation was arranged at three-month intervals. After six months of surveillance, the lesion has remained stable without progression.

**Follow-up and outcome of interventions:** the patient is followed in the outpatient clinic every three months for monitoring of cutaneous lesion changes. An annual chest and abdominal CT scan is performed to screen for metastatic disease. To date, the skin lesions have remained stable throughout the six-month follow-up period. Adherence to the surveillance protocol was verified via telephone questionnaire, with no missed appointments documented. No adverse events were reported during follow-up.

**Patient perspective:** the patient expressed understanding of the diagnosis and the recommended treatment of wide local excision. However, she declined surgical intervention primarily due to concerns about potential facial scarring and cosmetic impact. She agreed to regular clinical monitoring for any changes in the lesion.

**Informed consent:** written informed consent was obtained from the patient for the publication of this case report.

## Discussion

The strength of this case report lies in its detailed characterization of a primary cutaneous epithelioid hemangioendothelioma arising at a rare site, highlighting the essential role of histopathological examination and immunohistochemistry in achieving an accurate diagnosis. Furthermore, obtaining an early histopathological diagnosis is critical for guiding treatment selection. When diagnosed while the lesion is still small, complete surgical excision can be performed, thereby addressing both therapeutic and cosmetic objectives. A limitation of this case is the inability to perform molecular testing due to platform constraints, which precluded further genotypic characterization. Moreover, the follow-up duration has been insufficient to evaluate disease progression and long-term prognosis under non-surgical management. cEHE is frequently misdiagnosed as other epithelioid lesions in clinical practice, including epithelioid angiosarcoma, conventional epithelioid sarcoma, and epithelioid hemangioma [4]. Among these, epithelioid angiosarcoma, a high-grade mimic, is characterized by significant cytologic atypia and high mitotic activity. Epithelioid sarcoma typically demonstrates cytokeratin expression and lacks ERG immunoreactivity. In contrast, epithelioid hemangioma displays well-formed vascular channels and a prominent inflammatory infiltrate. Key diagnostic features, such as infiltrative growth, epithelioid morphology, and intracytoplasmic lumina, together with a characteristic immunoprofile (CD31+/ERG+/CD34+), are critical for confirming cEHE. The notably elevated Ki-67 proliferation index of 30% represents an atypical feature in this case. Conventional EHE typically demonstrates low proliferative activity, with Ki-67 indices generally below 10%. A higher index, as observed here, may correlate with more aggressive biological behavior.

Notably, approximately 90% of EHE cases harbor a WWTR1-CAMTA1 gene fusion [5], while a minority harbor a YAP1-TFE3 fusion [6]. These genetic alterations can be confirmed by fluorescence in situ hybridization or next-generation sequencing, which are particularly valuable in morphologically ambiguous cases.

The standard management for localized cEHE is complete surgical excision with clear margins [7]. However, as demonstrated here, tumor location can pose considerable therapeutic challenges. In cosmetically sensitive areas such as the face, patients may be reluctant to undergo extensive resection due to functional and aesthetic concerns. This highlights the importance of individualized treatment planning and thorough discussion of the risks, including local recurrence and metastasis, and the benefits of each therapeutic option.

For patients with inoperable lesions or those who decline surgery, radiotherapy or systemic therapies, particularly anti-angiogenic agents, may be considered, although evidence supporting their efficacy in cEHE remains limited [7]. Several such agents, including interferon-α, sirolimus, thalidomide, sorafenib, and bevacizumab, have been associated with partial responses or prolonged disease stabilization in this setting [8]. Additionally, given the established role of the WWTR1-CAMTA1 gene fusion in activating the Hippo-YAP/TAZ signaling pathway, the MEK inhibitor trametinib has shown promising antitumor effects in preclinical models and may represent a future targeted therapeutic strategy [8].

Epithelioid hemangioendothelioma (EHE), while associated with a more favorable prognosis than angiosarcoma, still carries a significant risk of local recurrence following surgical resection. When occurring in the head and neck region, EHE demonstrates a local recurrence rate of 44% and a metastasis rate of 38% [9]. Therefore, long-term clinical follow-up is strongly recommended for all patients, including those managed conservatively.

## Conclusion

This case emphasizes that primary cEHE can occur on the head and neck. Pathologists and dermatologists should be familiar with its features to ensure timely and accurate diagnosis. Treatment should be patient-centered, balancing oncologic principles with cosmetic outcomes, and should emphasize the importance of long-term surveillance.
